# The Diagnostic Value of Serum C-Reactive Protein for Identifying Pneumonia in Hospitalized Patients with Acute Respiratory Symptoms

**DOI:** 10.1155/2016/2198745

**Published:** 2016-08-16

**Authors:** Agustín Ruiz-González, Laia Utrillo, Silvia Bielsa, Miquel Falguera, José M. Porcel

**Affiliations:** Department of Internal Medicine, Arnau de Vilanova University Hospital, Biomedical Research Institute of Lleida Foundation Dr. Pifarré (IRBLleida), 25198 Lleida, Spain

## Abstract

*Background*. The clinical diagnosis of pneumonia is sometimes difficult since chest radiographs are often indeterminate. In this study, we aimed to assess whether serum C-reactive protein (CRP) could assist in identifying patients with pneumonia.* Methods*. For one winter, all consecutive patients with acute respiratory symptoms admitted to the emergency ward of a single center were prospectively enrolled. In addition to chest radiographs, basic laboratory tests, and microbiology, serum levels of CRP were measured at entry.* Results*. A total of 923 (62.3%) of 1473 patients hospitalized for acute respiratory symptoms were included. Subjects with a final diagnosis of pneumonia had higher serum CRP levels (median 187 mg/L) than those with exacerbations of chronic obstructive pulmonary disease (63 mg/L) or acute bronchitis (54 mg/L, *p* < 0.01). CRP was accurate in identifying pneumonia (area under the curve 0.84, 95% CI 0.82–0.87). The multilevel likelihood ratio (LR) for intervals of CRP provided useful information on the posttest probability of having pneumonia. CRP intervals above 200 mg/L were associated with LR+ > 5, for which pneumonia is likely, whereas CRP intervals below 75 mg/L were associated with LR < 0.2, for which pneumonia is unlikely.* Conclusion*. Serum CRP may be a useful addition for diagnosing pneumonia in hospitalized patients with acute respiratory symptoms.

## 1. Introduction

Pneumonia is a leading cause of hospitalization and death in developed countries [1]. However, the discrimination of pneumonia from other lower respiratory tract infections (LRTI), where antibiotics are not required, is sometimes challenging, particularly in its early stages. In elderly patients, the clinical presentation is often nonspecific and interpreting chest radiographs can be difficult in patients with severe or previous pulmonary disease [2, 3].

Recent guidelines and review studies have suggested that serum C-reactive protein (CRP) may be helpful in distinguishing pneumonia from other acute respiratory illnesses [4, 5]. Nevertheless, the strength of this assertion is moderate as it is based on just a few previous studies whose designs were mainly retrospective and only included a small number of selected patients (e.g., chronic obstructive pulmonary disease (COPD) was sometimes excluded) [6–8].

A prospective study was therefore conducted in a large and unselected population with the goal of clarifying whether serum CRP could identify patients with pneumonia.

## 2. Patients and Methods

### 2.1. Subjects

A prospective study was performed in a 500-bed university hospital, and patients with the following inclusion criteria were recruited during one winter season (2013-14): (1) adults > 18 years old admitted to the emergency ward, (2) respiratory symptoms (cough, sputum production, dyspnea, tachypnea, and pleuritic pain) as the main complaint, with or without fever, and (3) disease duration of less than two weeks. The exclusion criteria were (1) a final diagnosis of acute decompensated heart failure, pulmonary embolism, lung cancer, or an upper respiratory infection (e.g., acute pharyngitis, rhinitis, and sinusitis), (2) severe immunosuppression (e.g., human immunodeficiency virus infection and hematological diseases) or receiving immunosuppressive therapy (i.e., prednisone or an equivalent dose of 15 mg daily for 2 weeks or other immunosuppressant drugs), and (3) no hospitalization required.

Hospitalization was considered necessary if patients met one of the following: (1) need for either respiratory support (Sa 02 < 90% or Pa 02/Fi 02 < 300), mechanical ventilation (respiratory acidosis with pH < 7.30), or vasopressor drugs, (2) worsening of associated comorbidities (e.g., decompensated heart failure), (3) inability to take oral drugs, or (4) no response to an initial adequate treatment in the emergency department.

The local ethics committee approved this study and written informed consent was obtained from each patient.

### 2.2. Measurements

At the initial visit to the emergency department, demographic and basic clinical information was collected from each patient. In addition to routine blood tests, a serum sample was obtained to measure CRP. Microbiological studies included sputum sampling for Gram staining and culture in all patients with LRTI, when possible, as well as blood cultures, and* Streptococcus pneumoniae* and* Legionella pneumophila* antigen detection tests in urine samples from those with pneumonia. Serology was ordered according to the criterion of the attending physician. To stratify severity in pneumonia patients, a validated prediction rule was used, namely, the CRB65 Severity Index [9]. Antibiotic therapy was administered in the emergency department based on the clinician's judgment.

Blood samples for CRP were analyzed by a particle-enhanced turbidimetric assay following the manufacturer's instructions (Beckman Coulter, USA). The range of detection for this CRP assay is from 0.2 to 480 mg/L.

### 2.3. Disease Criteria

LRTI was defined by the presence of at least one respiratory symptom (e.g., cough, sputum production, dyspnea, tachypnea, and pleuritic pain) plus at least one finding during auscultation (i.e., crackles) or one sign of infection (temperature > 38°C, shivering, leukocyte count >10, or <4 × 10^9^ cells), regardless of antibiotic use. For pneumonia, a new infiltrate on the chest radiograph was also required. COPD was defined by postbronchodilator spirometric criteria, according to the Global Initiative for Chronic Obstructive Lung Disease (GOLD) guidelines, as FEV_1_/FVC ratio < 70%. Acute bronchitis was defined as LRTI in the absence of an underlying lung disease (COPD) or focal chest infiltrates on chest X-rays [10, 11]. The chest X-rays were reviewed by two clinicians with expertise in chest infections.

The diagnosis of heart failure was made on clinical grounds (history, physical examination, chest radiograph, electrocardiogram, echocardiogram, and response to diuretic treatment), according to the American College of Cardiology/American Heart Association guidelines [12]. Pulmonary embolism was the final diagnosis when intraluminal filling defects were observed in computed tomographic pulmonary angiography.

### 2.4. Statistical Analysis

Results are reported as means (SD) or medians (quartiles) as appropriate. Comparisons between groups were performed with *χ* ^2^ and Fisher's exact tests for categorical variables and the nonparametric Kruskal-Wallis and Mann-Whitney *U* tests for continuous variables. Sensitivity, specificity, and positive and negative likelihood ratios (LR), with confidence intervals based on exact binomial distribution, were calculated using standard methods. The area under the receiver operating characteristic curve (AUC) was used to establish the optimum cut-off points for CRP and leukocyte counts. Multilevel LRs were calculated as previously described with the use of equally spaced cut-off points [13]. Statistical significance was established at *p* ≤ 0.05. Calculations were performed with statistical software SPSS version 22.0 (Chicago, IL, USA).

## 3. Results and Discussion

### 3.1. Results

A total of 1473 consecutive patients admitted to the emergency ward with acute respiratory symptoms were initially recruited, of whom 550 were excluded because of diagnoses other than LRTI (309), immunosuppressive condition or therapy (52), or no hospitalization requirement (189). Therefore, 923 patients with the final diagnoses of pneumonia (557) or other LRTI (366), namely, acute bronchitis and acute exacerbation of COPD, were included (Table 1).

Patients with pneumonia had a median CRB65 score of 2 (IQR, 1–5) and microorganisms were found in 171 (30.7%), as follows:* Streptococcus pneumoniae* (118),* Haemophilus influenzae* (23),* Chlamydophila pneumoniae* (7),* Legionella pneumophila* (5), influenza A (5),* Pseudomonas aeruginosa* (4),* Mycobacterium tuberculosis* (4),* Mycoplasma pneumoniae* (2), and one for each of* Moraxella catarrhalis*,* Staphylococcus aureus, Escherichia coli, and Enterococcus faecium*. In comparison with other LRTI, patients with pneumonia were younger and had fewer comorbid conditions, higher temperatures, and both higher blood leukocyte counts and serum CRP levels. Additionally, more patients with pneumonia required admission to the intensive care unit, although in-hospital mortality was similar between groups (Table 1).

In a logistic regression model, only 4 variables were independently related to pneumonia diagnosis: under 70 years of age (OR 2.83; 95% CI 1.95–4.09), temperature > 38°C (OR 2.51, 95% CI 1.65–3.81), leukocyte count > 15 × 10^9^/L (OR 2.21, 95% CI 1.5–3.25), and serum CRP > 150 mg/L (OR 10.44, 95% CI 7.24–15.05).

**Figure 1:**
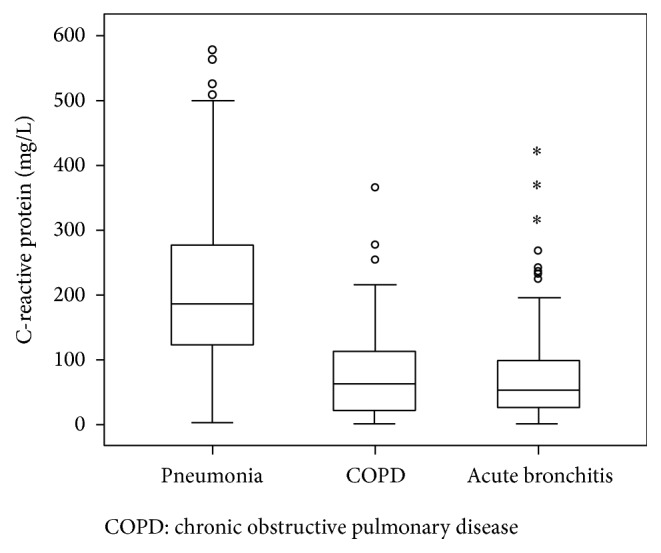
C-reactive protein levels in the study population. *∗* represents extreme values.

Serum CRP levels according to disease etiologies are shown in Figure 1. Subjects with pneumonia had higher serum CRP concentrations (median 187 mg/L) than those with exacerbations of COPD (63 mg/L) or acute bronchitis (54 mg/L, *p* < 0.01). The CRP reached AUC of 0.84 (95% CI 0.82–0.87) to distinguish pneumonia from other LRTI. The operating characteristics of different cut-off serum CRP values are shown in Table 2. For example, a serum CRP > 200 mg/L identified pneumonia with a sensitivity, specificity, and positive and negative LR of 44.8%, 95.6%, and 10.2 and 0.5, respectively. Moreover, the positive LR for several CRP intervals was calculated (Table 3). Thus, CRP intervals above 200 mg/L were associated with LR positive greater than 5 (for which pneumonia is likely), whereas CRP intervals below 75 mg/L were associated with LR lower than 0.2 (for which pneumonia is unlikely).

It was also observed that CRP levels were not related to the variable “days of symptoms.” Indeed, median CRP values in patients with ≤2, 3–5, and ≥6 days of symptoms were 107 mg/L (45–196), 127 mg/L (45–214), and 123 mg/L (43–222), respectively (*p* = 0.70).

### 3.2. Discussion

This study showed that serum CRP measurements upon admission to the hospital are useful for distinguishing patients with pneumonia from those with other LRTI.

Previous studies have investigated the utility of serum CRP in identifying pneumonia. In a retrospective analysis of 60 patients with LRTI, 75% of patients with pneumonia had serum CRP levels > 100 mg/L, although no information was reported on specificity [6]. In a prospective study of 97 patients with pneumonia, it was found that only 5% had serum CRP levels below 50 mg/L [7]. In another prospective study of 284 patients with LRTI, a serum CRP > 100 mg/L had a specificity of 96% for labeling pneumonia [8], even though patients with acute exacerbation of COPD were excluded from the analysis. Based on these studies, the British Thoracic Society stated that the measurement of serum CRP on admission may be helpful in distinguishing pneumonia from other LRTI, with moderate weight being placed on this recommendation [4].

More recently, in a post hoc analysis of 545 patients with LRTI, Müller et al. [14] found that serum CRP had AUC of 0.76 in identifying patients with pneumonia. However, only 11% of patients included had acute exacerbation of COPD, and the technique used (a highly sensitive CRP) is not widely available in clinical practice. Finally, Bafadhel et al. [15] studied 158 patients with LRTI and concluded that a cut-off point for serum CRP of >48 mg/L had a sensitivity and specificity of 91% and 93% for pneumonia, respectively. Even so, patients with acute exacerbations of COPD were also excluded from this analysis.

Previous studies usually recommended a single CRP cut-off point to dichotomize respiratory infections into either pneumonia or nonpneumonia categories. This approach eliminates much of the diagnostic information contained in laboratory tests that have continuous integer values. An alternative strategy for improving the discriminative properties of diagnostic tests is to generate multilevel LRs using various cut-off points and then apply them to convert the pretest probabilities into posttest probabilities of having pneumonia [16]. This new strategy, when applied to laboratory results in the borderline pneumonia range with the use of single cut-off points, generates low LRs that will not misclassify patients if the pretest suspicion for pneumonia is low. Medical literature commonly describes the operating characteristics of a diagnostic test by dichotomizing test results into normal and abnormal values and calculating their sensitivity and specificity. Unfortunately, this knowledge offers little clinical utility when evaluating individual patients because these indexes do not describe the probability of disease if the result is positive or negative, as LR does. Moreover, the rationale behind the use of multilevel LRs is that the dichotomization of test results does not assist in assessing to what degree a test result alters a clinician's estimation of the pretest probability of disease [13].

In our study, we sought to improve upon the shortcomings of the previous ones. First, this is the largest study performed on this issue to date. Second, the population was derived from unselected patients admitted to the emergency department with acute respiratory symptoms, including those with acute exacerbations of COPD. Third, the design of the study was prospective. Finally, the multilevel LRs for several intervals of serum CRP provided useful information on the posttest probability of having pneumonia. Thus, serum CRP intervals above 200 mg/L were associated with LR positive > 5 (for which pneumonia is likely), whereas CRP intervals below 75 mg/L were associated with LR < 0.2 (for which pneumonia is unlikely). Between these intervals, the serum CRP did not provide useful clinical information.

One drawback of the study was that detailed information on physical signs was not provided. However, previous studies have shown that there are no findings from the history or physical examination capable of confidently ruling in or out the diagnosis of pneumonia [2]. Also, other potentially useful biomarkers of infection were not tested. For instance, procalcitonin has been reported to have high discriminative power in identifying pneumonia [17] although it is not widely available in emergency settings.

## 4. Conclusion

The results of our study suggest that the routine use of serum CRP levels in hospitalized patients with acute respiratory symptoms can help clinicians to differentiate pneumonia from other respiratory infections. Indeed, serum CRP levels above 200 mg/L or below 75 mg/L make the diagnosis of pneumonia likely or unlikely, respectively. A further prospective validation of CRP ranges in an independent population is warranted.

## Figures and Tables

**Table 1:** Baseline characteristics of patients admitted with lower respiratory tract infections.

	Pneumonia (*n* = 557)	Other lower respiratory tract infections^*∗*^ (*n* = 366)	*p* value
*Demographics*			
Age, years	72 (56–80)	79 (71–85)	<0.01
Gender, male	349 (63)	232 (54)	0.84
Comorbidity (Charlson index)	4 (2–6)	6 (4–7)	<0.01

*Clinical variables*			
Days of symptoms	4 (2–7)	4 (2–7)	0.27
Previous antibiotic treatment	164 (33)	100 (30)	0.40
Heart rate (bpm)	98 (84–110)	97 (84–110)	0.56
Respiratory rate (rpm)	28 (24–32)	28 (24–32)	0.79
Systolic blood pressure (mmHg)	123 (109–140)	133 (116–146)	<0.01
Diastolic blood pressure (mmHg)	69 (60–78)	71 (63–82)	<0.01
Temperature (°C)			
No fever (<37°C)	197 (35)	219 (60)	<0.01
Low-grade fever (37-38°C)	163 (29)	87 (24)	
High-grade fever (>38°C)	197 (35)	60 (16)	

*Laboratory findings*			
Basal pO_2_(mmHg)	61 (55–70)	62 (55–72)	0.19
C-reactive protein (mg/L)	187 (123–278)	59 (24–108)	<0.01
Leukocyte count (×10^9^/L)	13.3 (9.27–17.65)	10.8 (7.97–13.30)	<0.01
Creatinine (mg/dL)	1.0 (0.8–1.3)	0.9 (0.7–1.2)	0.01

*Microbiology findings*			
Microorganism found	169 (30.3%)	30 (8.9%)	<0.01

*Follow-up*			
Days in hospital	6 (4–10)	6 (4–9)	0.05
Intensive care unit transfers	37 (7)	4 (1)	<0.01
In-hospital mortality	31 (6)	16 (4)	0.45

Quantitative variables are shown as medians (IQR 25–75) and qualitative variables as absolute numbers (percentages). ^*∗*^Other lower respiratory tract infections included acute bronchitis and acute exacerbations of COPD.

**Table 2:** Operating characteristics of C-reactive protein for identifying pneumonia according to different serum values.

Serum CRP (mg/L)	Sensitivity, % (95% CI)	Specificity, % (95% CI)	LR+	LR−
≥50	91.3 (88.7–93.4)	43.9 (38.9–49.1)	1.6 (1.4–1.7)	0.2 (0.1–0.2)
≥100	82.4 (79.0–85.3)	72.3 (67.6–76.7)	3 (2.5–3.5)	0.2 (0.2-0.2)
≥150	65.1 (61.1–69.0)	87.16 (83.2–90.2)	5.0 (3.8–6.6)	0.4 (0.3–0.4)
≥200	44.8 (40.8–49.0)	95.6 (93.0–97.2)	10.2 (6.3–16.6)	0.5 (0.5–0.6)

CRP, C-reactive protein; LR, likelihood ratio.

**Table 3:** Multilevel likelihood ratios for different serum C-reactive protein intervals.

Serum CRP (mg/L)	Pneumonia (*n* = 557)	Other lower respiratory tract infections^*∗*^ (*n* = 366)	LR+
>250	171	7	16.3 (7.7–34.4)
225–250	40	5	5.3 (2.1–13.4)
200–225	32	4	5.3 (1.9–15.0)
175–200	53	17	2.0 (1.2–3.5)
150–175	58	13	2.9 (1.6–5.3)
125–150	46	23	1.3 (0.8–2.1)
100–125	49	30	1.1 (0.7–1.6)
75–100	31	42	0.5 (0.3–0.7)
50–75	19	60	0.2 (0.1–0.3)
25–50	27	67	0.2 (0.1–0.4)
<25	20	98	0.1 (0.0–0.2)

CRP, C-reactive protein; LR, likelihood ratio.

^*∗*^Other lower respiratory tract infections included acute bronchitis and acute exacerbations of COPD.
